# Predicting Motif-Mediated Interactions Based on Viral Genomic Composition

**DOI:** 10.3390/ijms26083674

**Published:** 2025-04-13

**Authors:** Sobia Idrees, Keshav Raj Paudel, Mithila Banik, Newton Suwal, Rajan Thapa, Saroj Bashyal

**Affiliations:** 1 School of Biotechnology and Biomolecular Sciences, University of New South Wales, Sydney, NSW 2033, Australia; 2 Centre for Inflammation, Centenary Institute and the University of Technology Sydney, School of Life Sciences, Faculty of Science, Sydney, NSW 2007, Australia; 3 Department of Bioinformatics and Biotechnology, Asian University for Women, Chittagong 4000, Bangladesh; 4 Department of Pharmacy, Manmohan Institute of Health Sciences, Tribhuvan University, Kathmandu 44600, Nepal; 5 Department of Pharmacy, Universal College of Medical Sciences, Tribhuvan University, Bhairahawa, Rupendehi 32900, Nepal

**Keywords:** virus–host interactions, bioinformatics, viral mimicry, short linear motifs

## Abstract

Viruses manipulate host cellular machinery to propagate their life cycle, with one key strategy being the mimicry of short linear motifs (SLiMs) found in host proteins. While databases continue to expand with virus–host protein–protein interaction (vhPPI) data, accurately predicting viral mimicry remains challenging due to the inherent degeneracy of SLiMs. In this study, we investigate how viral genomic composition influences motif mimicry and the mechanisms through which viruses hijack host cellular functions. We assessed domain–motif interaction (DMI) enrichment differences, and also predicted new DMIs based on known viral motifs with varying stringency levels, using SLiMEnrich v.1.5.1. Our findings reveal that dsDNA viruses capture significantly more known DMIs compared to other viral groups, with dsRNA viruses also exhibiting higher DMI enrichment than ssRNA viruses. Additionally, we identified new vhPPIs mediated via SLiMs, particularly within different viral genomic contexts. Understanding these interactions is vital for elucidating viral strategies to hijack host functions, which could inform the development of targeted antiviral therapies.

## 1. Introduction

The evolutionary arms race between viruses and their hosts drives continuous adaptation in viral molecular mechanisms and host defense systems. Viruses evolve rapidly to evade host immunity, while hosts develop countermeasures to detect and neutralize infections [1,2,3]. Central to this battle are virus–host protein–protein interactions (vhPPIs), which facilitate viral pathogenesis through receptor binding, membrane remodeling, and the hijacking of cellular machinery for replication and virion assembly [4]. A significant subset of vhPPIs is mediated via short linear motifs (SLiMs), which are conserved protein sequences (3–10 amino acids) that drive transient interactions in disordered protein regions [3,5,6,7]. These motifs enable viruses to hijack host processes through molecular mimicry, disrupting signaling, protein degradation, and immune responses [8,9]. Domain–motif interactions (DMIs) are mediated through SLiMs binding to host proteins via structural domains and are significantly more prevalent in vhPPIs, highlighting their importance in viral adaptation. SLiMs evolve rapidly, allowing viruses to adapt efficiently with minimal genomic investment [3]. SLiMs exhibit remarkable evolutionary plasticity, frequently arising or disappearing through mutations, shaping viral tropism and pathogenicity [1,10,11]. Convergent evolution has led to similar SLiMs emerging independently in unrelated viruses, underscoring their role in host adaptation [12]. Unlike eukaryotic SLiMs, viral motifs often display reduced disorder, allowing them to mimic both structured and unstructured host motifs [9]. Viral SLiMs often mimic host motifs, competing for critical regulatory sites in pathways like cell cycle control, immune signaling, and DNA repair [13,14]. Curated resources like the Eukaryotic Linear Motif (ELM) database aid in studying their role in viral pathogenesis [15]. Intrinsically disordered regions in viral proteomes are enriched in SLiMs, facilitating molecular mimicry. An analysis of 2278 viral genomes revealed disorder levels between 2.9% and 23.1%, independent of genome size [16,17]. A broader study of 6108 viral proteomes found that ssRNA-RT viruses had the highest disorder while dsRNA viruses had the lowest [18]. Disorder patterns also varied by host organism, suggesting adaptive specialization. Recent studies further illustrate the dynamic nature of viral SLiM evolution. In SARS-CoV-2, transient SLiMs emerge through mutations, covering ~25% of known eukaryotic motifs and demonstrating the virus’s ability to exploit host interactions [19]. Similarly, subtype-specific variations in HIV-1 Gag protein [20] and structural differences in influenza A haemagglutinin [21] highlight how genetic diversity influences host interactions and immune evasion. Comparative analyses reinforce the modular organization of viral proteins in host adaptation. Functional domains in coronavirus non-structural polyproteins [22] and phenotype-specific proteomes in baculoviruses [23] show how viruses tailor interactions for efficient host exploitation.

While previous studies have examined RNA and DNA viruses, none have explored differences at the level of single- and double-stranded genomes. However, a systematic analysis of SLiM-mediated DMIs across viral genomes remain lacking [6,12,24]. This study aimed to identify new SLiM-mediated interactions and determine whether viruses employ distinct molecular mimicry strategies based on their genomic composition. The findings reveal how viral genome composition influences SLiM utilization, providing insights into host disruption mechanisms and antiviral development.

## 2. Results

**Figure 1:**
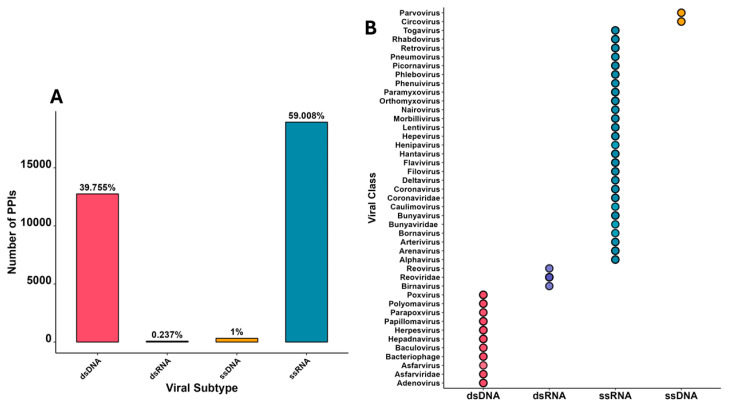
Protein interactions in PHISTO database categorized based on viral genomic composition, (**A**) proportion of vhPPIs in each viral genomic category, (**B**) viral classes in each viral genomic category.

The vhPPI data used in this study were obtained from the PHISTO database [retrieved on 10 December 2024], containing 42,116 PPIs from 474 distinct viral strains, which were filtered to include only viruses with DNA or RNA genomes. These viruses were further classified into four genomic categories where single-stranded RNA (sRNA) had the highest proportion of vhPPIs (~59% vhPPIs), double-stranded RNA (dsRNA: ~0.24% vhPPIs), single-stranded DNA (ssDNA: ~1% vhPPIs), and double-stranded DNA (dsDNA, ~40% vhPPIs) (Figure 1A). The dsDNA category included 12 viral classes consisting of 173 viral strains, while the dsRNA category contained 3 viral classes having 13 viral strains, ssDNA had 2 classes with 4 viral strains, and ssRNA had 27 viral classes consisting of 284 viral strains (Figure 1B). To ensure data quality, only reviewed UniProt proteins were kept, and redundant PPIs were removed, leaving only unique interactions in the dataset. This final dataset, reflecting a diverse array of viral interactions across genomic classes, was used for subsequent analyses to evaluate enrichment and predict new interactions.

### 2.1. Enrichment of DMIs Based on Viral Genomic Composition

In our analysis, we observed that PHISTO had a higher number of ssRNA interactions compared to dsRNA interactions and, similarly, more dsDNA interactions than ssDNA interactions. To assess whether DNA or RNA viruses were enriched for DMIs, we performed a DMI enrichment analysis using the *SLiMEnrich v1.5.1* [25] tool with the ELMi-Protein stringency, which calculates the enrichment of DMIs in a PPI dataset.

It was observed that dsRNA viruses exhibited the highest enrichment for DMIs, with ~19x enrichment compared to random, followed by dsDNA (E-score: ~11), and ssRNA (E-score: 6.7). This showed that these viral groups were indeed capturing DMIs and thus can be used in studying molecular mimicry in viruses (Table 1). In contrast, ssDNA viruses did not capture any known DMIs, likely due to a lack of known interactions. As a result, ssDNA viruses were excluded from further analysis, as their lack of DMI enrichment limited their relevance for evaluating DMIs in this study. Overall, the number of known DMIs in these viral groups was relatively low, likely because only a few viral DMIs (~132) have been reported to date in the ELM database.

### 2.2. DMI Prediction Using Known Viral ELMs

Once it was established that vhPPIs of dsDNA, dsRNA, and ssRNA viruses were capturing DMIs, the next step was to apply the more complex and noisier ELMc-Protein strategy, where known viral mimicry instances were used to predict additional DMIs. This approach aimed to increase the number of predicted DMIs and identify new interactions mediated via known mimicry candidates. In this study, we focused exclusively on known mimicry candidates to predict DMIs, given the high FDR associated with interactions of this nature. For the ssRNA viruses, there were 49 potential DMIs and 9 predicted DMIs, resulting ~8.2x enrichment. dsDNA viruses had 87 potential DMIs and 38 predicted DMIs, with a ~12x enrichment. In contrast, dsRNA viruses had 3 potential DMIs and 3 predicted DMIs, yielding an enrichment score of 16.22. This highlights the need to identify new DMIs that could aid in studying molecular mimicry by viruses. The high enrichment (*p* < 0.05) of additional predicted DMIs suggests their likelihood of being real. Human papillomavirus (HPV), especially types 16 and 18, was identified as the most common virus exploiting host proteins through DMIs. HPV may be more involved in DMIs due to its ability to hijack human proteins through these interactions, allowing it to persist in the host and evade the immune system. Following HPV, Bluetongue virus, from the dsRNA group, was also prominently represented, indicating its significant involvement in DMIs. Sindbis virus, from the ssRNA group, was also identified as a contributor, though with lesser frequency. Other viruses, such as Epstein–Barr virus (EBV), human herpesvirus 1 (HHV-1), human adenovirus types C and D, Hepatitis C, Influenza A, etc., were present but to a lesser extent. These findings suggest that HPV might more commonly interact with human proteins through DMIs (Table S1).

### 2.3. Expanding the DMI Network Through Incorporating Domain Information

**Figure 2:**
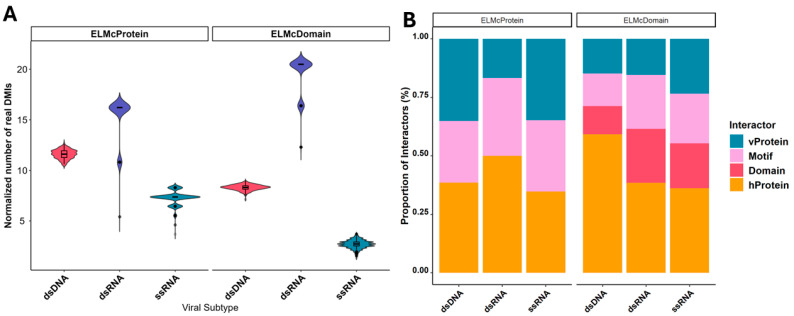
DMI enrichment using different stringencies, (**A**) normalized number of real DMIs (DMI Real = DMI Obs − DMI Ran). Real DMIs captured by the different viral groups available in PHISTO database over 1000x randomizations using different strategies. *Y*-axis shows the normalized number of real DMIs, (**B**) Proportion of interacting proteins, motifs, and domains captured by each viral category using different stringencies.

To enhance the predicted DMI count, a more stringent SLiMEnrich setting (ELMc-Domain) was applied. This approach linked known viral instances to human proteins via Pfam domains. The normalization of the data was achieved by dividing the number of real DMIs by mean random DMIs. The ELMc-Domain strategy maintained a modest FDR, suggesting that even noisier DMI predictions might still return a lot of real DMI. The analysis identified 111 predicted NR DMIs in dsDNA, exhibiting an enrichment of 9.3. These results were highly significant (*p* < 0.001), but the FDR was 0.1, suggesting that ~10% of these predictions might be false positives. In the case of dsRNA, five predicted DMIs were identified, exhibiting a strong E-score of 20.4 and demonstrating significant statistical relevance (*p* < 0.001, FDR < 0.04). This indicates a reliable signal, suggesting that the analysis captured a higher number of real DMIs (Figure 2A). In comparison, ssRNA showed 19 predicted NR DMIs with enrichment of 3.69, but the FDR was 0.2, pointing to an increased risk of false positives in this category (Supplementary Table S2).

On a broader note, across all viral genomic categories, the analysis revealed that fewer viral proteins were hijacking around 2x human proteins. Specifically, for double-stranded viruses, the analysis showed that only a small number of viral proteins were interacting with a considerably larger number of human proteins. For instance, dsDNA viruses had 16 viral proteins interacting with 64 human proteins, dsRNA had 2 viral proteins interacting with 5 human proteins, and ssRNA had 11 viral proteins interacting with 17 human proteins. This suggests that these viral proteins may have the ability to mimic a broad spectrum of human proteins, potentially allowing them to hijack the host’s cellular machinery for various functions (Figure 2B).

### 2.4. Host Proteins Targeted by Different Viral Genomic Categories

We investigated the distribution of hijacked host proteins across different viral genome types to determine whether certain proteins were uniquely targeted by one viral category or shared among multiple categories. Our analysis revealed that most hijacked host proteins were associated with a single viral type, with fewer proteins being shared across categories.

Among the uniquely targeted proteins, dsDNA viruses hijacked the largest number, with 61 proteins identified. ssRNA viruses targeted 10 unique host proteins, while dsRNA viruses had only 1 uniquely targeted protein. This suggests that dsDNA viruses may rely on a broader range of host interactions compared to the other viral types.

In addition to unique associations, we examined proteins that were shared between pairs of viral genome types. The dsRNA + dsDNA group included 3 host proteins that were targeted by both viral types. Similarly, the dsRNA + ssRNA group shared 2 hijacked proteins, and the dsDNA + ssRNA group had 6 host proteins in common. Notably, only one host protein, NEDD4, was found to be commonly hijacked by all three viral genome types—dsRNA, dsDNA, and ssRNA (Table 2).

These findings show that, while the majority of viral hijacking events appear specific to a single genome composition, a subset of host proteins is targeted by multiple viral groups. This pattern suggests that certain host factors may play central roles in viral replication strategies shared across different virus families.

### 2.5. Pathways Hijacked by Different Viral Groups

**Figure 3:**
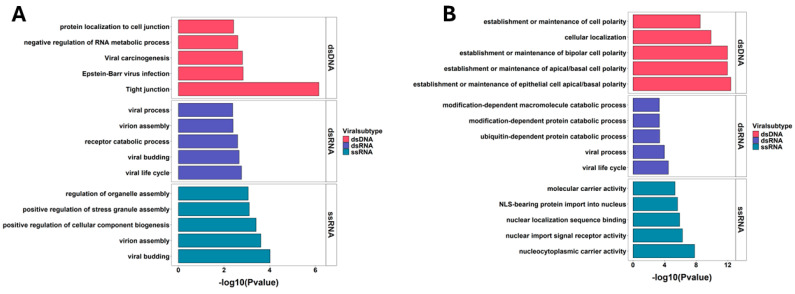
Biological pathways hijacked by human protein interactors, (**A**) pathways regulated by human proteins predicted to interact with viral proteins via SLiMs using ELMc-Protein stringency, (**B**) pathways regulated by human proteins predicted to interact using their domains with viral proteins via SLiMs using ELMc-Domain stringency.

The viral groups—dsDNA, dsRNA, and ssRNA viruses—hijacked key cellular pathways to facilitate their replication and survival. dsDNA viruses disrupted pathways related to tight junctions, Epstein–Barr virus infection, viral carcinogenesis, RNA metabolism, and protein localization to cell junctions. This interference likely compromised cellular integrity and gene expression regulation, creating an environment conducive to viral replication and potentially contributing to oncogenesis. dsRNA viruses targeted processes such as the viral life cycle, virion assembly, receptor catabolic processes, and viral budding, indicating the manipulation of host cell machinery to support viral replication and release. ssRNA viruses, in turn, hijacked pathways involved in viral budding, virion assembly, regulation of stress granule assembly, and organelle biogenesis, suggesting alterations in cellular responses to stress and the modulation of cellular structures for efficient viral propagation. These disruptions of cellular pathways highlighted the complex interactions between viruses and host cells, essential for viral pathogenesis, and could offer insights into potential therapeutic targets (Figure 3A).

All viral groups hijacked several critical cellular pathways, with human proteins interacting through Pfam domains and viral SLiMs predicted via the ELMc-Domain stringency analysis. dsDNA viruses disrupted processes related to the establishment and maintenance of epithelial cell polarity (apical/basal and bipolar), cell localization, and overall cell polarity. These disruptions likely altered host cell organization and function, providing a favorable environment for viral replication and survival. dsRNA viruses targeted key processes in the viral life cycle, viral processes, and protein catabolic pathways, such as ubiquitin-dependent and modification-dependent protein degradation. These viral interactions could modify host cell machinery, degrade essential proteins, and regulate cellular resources to support viral replication. ssRNA viruses manipulated pathways involved in nucleocytoplasmic transport, including nucleocytoplasmic carrier activity, nuclear import signal receptor activity, nuclear localization sequence binding, and the import of NLS-bearing proteins into the nucleus. This interference likely enhanced viral entry into the nucleus, facilitating replication. Overall, the predicted interactions between viral SLiMs and human proteins through Pfam domains highlight the strategic manipulation of host cell processes via viral subtypes, which is essential for viral pathogenesis and could offer insights into potential therapeutic targets (Figure 3B).

### 2.6. Cross-Validation of Predictions Using ELM Known Interactions

To evaluate enrichment, we calculated the proportion of known interactions identified in our predictions relative to the total number of predictions for each stringency condition within each viral group. Fisher’s exact test was then applied to assess whether the observed number of known interactions was significantly higher than expected by chance in each viral group. Under the ELMc-Protein stringency, dsDNA viruses had 31 known interactions, representing 79.4% of the total predictions. In contrast, under the ELMc-Domain condition, dsDNA viruses had 21 known interactions, accounting for 16.5% of total predictions. For dsRNA and ssRNA viruses, the number of known interactions remained consistent across both stringency conditions. dsRNA viruses had two known interactions (40.0%) under ELMc-Domain and two interactions (66.6%) under ELMc-Protein. Similarly, ssRNA viruses had six known interactions (31.5%) under ELMc-Domain and six interactions (66.6%) under ELMc-Protein.

**Figure 4:**
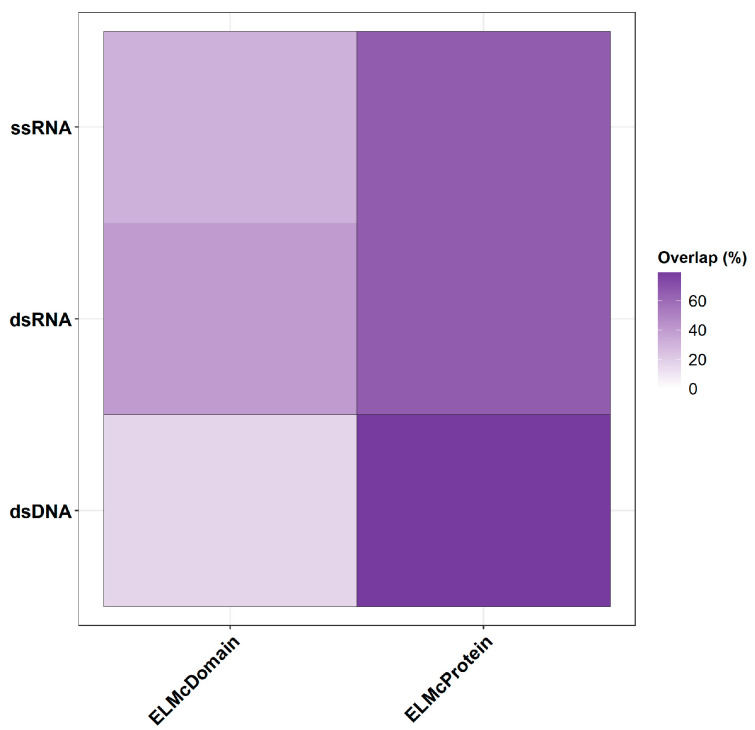
Proportion of known interactions in predicted DMIs from different stringencies.

The higher percentage of known interactions under the ELMc-Protein stringency suggests that these predictions are more likely to be accurate, as they align more closely with previously confirmed interactions in the ELM database. This increases the likelihood that other predicted DMIs might also be true interactions. The observed increase in known interactions under the more stringent prediction criteria, especially for dsDNA viruses, implies that additional predicted DMIs may indeed represent genuine interactions (Table 3, Table S2, Figure 4).

## 3. Discussion

This study was conducted to investigate how viruses with different genomic compositions manipulate host cellular machinery through motif mimicry and SLiM-mediated interactions. An analysis of the PHISTO database revealed a disparity in the availability of PPI data across different viral groups. ssRNA and dsDNA viruses had significantly more documented interactions compared to dsRNA and ssDNA viruses. This difference can be attributed to several factors: ssRNA viruses, such as coronaviruses and influenza, are responsible for major human diseases, leading to extensive research on their host interactions. In contrast, dsRNA viruses rarely infect humans, resulting in fewer studies. Similarly, ssDNA viruses, which have small genomes, rely heavily on host cellular machinery for replication, leading to fewer unique viral proteins and, consequently, fewer PPIs to analyze. Additionally, large-scale experimental approaches prioritize medically significant RNA viruses, further contributing to the limited data for dsRNA and ssDNA viruses [24].

To examine how different viruses manipulate host cellular machinery, DMI predictions were made for each viral genomic category. The first step was to evaluate whether these viruses were enriched for DMIs. In this study, SLiMEnrich v1.5.1 [25] was employed to assess DMI enrichment across various viral groups. Significant enrichment was observed for all groups except ssDNA viruses, for which no DMIs were predicted. This absence may be due to the limited number of available PPIs involving ssDNA viruses, which restricts the detection of potential DMIs. Additionally, ssDNA viruses typically possess small genomes, resulting in fewer viral proteins capable of interacting with host proteins. Another contributing factor could be the scarcity of known viral motifs within ssDNA viruses, further limiting DMI identification.

Next, an effort was made to predict new DMIs that might be biologically relevant using known viral SLiM instances and ELMc-Protein stringency of SLiMEnrich. Among the viral groups, dsDNA viruses captured the highest number of DMIs, followed by ssRNA and dsRNA viruses, with all predicted DMIs being ligand (LIG)-mediated. This indicates that dsDNA viruses have a greater ability to mimic host motifs and establish interactions with host proteins. Their large genome size and evolutionary adaptation may contribute to this, allowing them to encode multiple functional motifs. To further expand the network, ELMc-Domain stringency was used instead of alternative approaches due to the insufficiency of current known DMI/SLiM data [6,7,11]. However, since this strategy carries a higher risk of false positives, post-translational modifications (PTMs) (i.e., MOD and CLV) were excluded. Only four ELM types (LIG, DEG, DOC, and TRG) were included in the analysis to ensure meaningful predictions [11,12,26,27].

A comparison between RNA viruses (ssRNA and dsRNA) revealed that ssRNA vhPPIs captured more DMIs than dsRNA viruses. However, the FDR associated with ssRNA predictions (~0.2) was higher than that of dsRNA (~0.06), indicating that a greater proportion of ssRNA virus predictions may be false positives. Despite this, dsRNA viruses showed a greater enrichment trend in capturing DMIs, meaning that, although they had fewer interactions, those identified were more likely to be biologically relevant. This could be due to the relatively conserved nature of dsRNA virus–host interactions, which might be more functionally important despite their lower frequency.

Pathway enrichment analysis further revealed that human proteins targeted via ssRNA viral proteins were primarily involved in transport-related activities and were in the cytoplasmic region, where ssRNA viruses tend to replicate. This supports the notion that ssRNA viruses exploit the host’s transport machinery to facilitate viral replication and protein trafficking. Several key transport-related and cell-cycle regulation proteins, such as P53, ROA2, HNRPK, and NPM, have previously been reported as targets of RNA viruses [28,29]. In contrast, host proteins targeted via dsRNA viral proteins were mainly associated with catabolic and viral activity processes. This suggests that dsRNA viruses may rely more on host degradation pathways and viral replication machinery, which aligns with their replication strategy.

An analysis of DNA viruses (dsDNA and ssDNA) showed that dsDNA viruses had a significantly higher number of vhPPIs and were enriched for DMIs. This aligns with previous findings that dsDNA viruses, due to their larger genome size, encode more proteins that can interact with the host. Conversely, ssDNA viruses had very few vhPPIs and showed no enrichment for DMIs, leading to their exclusion from further analysis. The FDR for dsDNA predictions was ~0.1, indicating that, while some false positives were likely, a substantial number of predictions were reliable. GO enrichment analysis revealed that human proteins targeted by dsDNA viral proteins were primarily involved in localization-related processes, suggesting that these viruses manipulate host trafficking pathways to facilitate viral assembly and transport [24,30]. Previous studies have shown that RNA viruses are more likely than other viruses to target proteins involved in metabolic functions [31]. Additionally, a comparative analysis of DNA and RNA vhPPIs found that DNA viruses tend to target cellular and metabolic pathway proteins, whereas RNA viruses primarily interact with transport and metabolic proteins [32]. The findings from this study align with these observations, as most human proteins targeted by both RNA and DNA viruses were involved in transport-related processes, such as metabolic carrier activity. This suggests that, despite differences in genome structure and replication strategy, both RNA and DNA viruses have evolved to exploit host transport and metabolic systems for their survival.

The limited genomic resources of viruses exert evolutionary pressure to maximize their interactions with host proteins through DMIs to sustain their life cycle. One study found that viral proteins engage in more DMIs, contain more SLiMs than human proteins, and frequently mimic host proteins to enhance their survival [33]. Most of the viruses were interacting with approximately twice as many host proteins, a finding consistent with our previous study [12]. Given the high FDR of predictions from ELMc-Domain, it is likely that many predicted DMIs might be false positives. This may be due to the curation of data in PHISTO, which focuses on specific viruses or samples, potentially leading to biases in interaction data. These findings highlight the urgent need to develop more comprehensive vhPPI databases and improve the curation of vhPPI data to enhance the accuracy of interaction predictions. Expanding these databases with unbiased datasets covering a broader range of viral families could improve the reliability of DMI predictions and provide a more complete understanding of viral–host interactions.

## 4. Materials and Methods

### 4.1. Data Retrieval and Processing

A curated vhPPI dataset was obtained from the PHISTO database [retrieved on 10 December 2024] [34]. The dataset was categorized based on viral genomic composition, distinguishing between DNA and RNA viruses, as well as whether those were double- or single-stranded. Only interactions involving reviewed UniProt IDs were retained for analysis. We focused on all viruses that infect a variety of hosts, excluding bacteria. Additionally, experimentally validated SLiM data, known viral SLiM data, and known viral DMI data were retrieved from the ELM database [http://elm.eu.org/search.html (25 February 2025], a manually curated repository compiling SLiM occurrences from the literature [35]. A total of 327 SLiM classes, representing distinct motifs, along with 2278 experimentally confirmed protein instances and 200 associated interacting domains, were downloaded from the ELM database on 25 February 2025.

### 4.2. DMI Enrichment in Different Viral Groups

The downloaded ELM data were used to evaluate DMI enrichment and predict DMIs using vhPPI data. The sites for post-translational modification (MOD) and proteolytic cleavage site (CLV) ELM classes, which tend to have low complexity [11,34], were excluded from the analysis. These classes were excluded from the analysis to reduce the false discovery rate and focus on DMIs that are more likely to be true-positive by reducing noise in the network. Enrichment differences were evaluated using our previously published method, i.e., SLiMEnrich v1.5.1 [34], which explores a PPI network to identify pairs of proteins engaged in interaction, with the first protein either known or predicted to interact with the second protein through a DMI. Briefly, SLiMEnrich v1.5.1, employs three distinct strategies—ELMi-Protein, ELMc-Protein, and ELMc-Domain—to detect DMIs from interaction data using known viral SLiMs. Each strategy offers a different level of stringency: ELMi-Protein (the highest stringency) directly links motif-containing proteins to their domain-containing interaction partners without incorporating motif or domain information. ELMc-Protein (medium stringency) connects motif classes to known domain-containing protein partners while excluding domain information. ELMc-Domain (the lowest stringency) links motif classes to known interacting domains, offering the broadest search space. SLiMEnrich first identifies all potential DMI connections, which are then mapped onto PPI networks to pinpoint predicted DMIs within the dataset. Through permutation tests, it then assesses the count of known/predicted DMIs against the anticipated distribution under random association of the two protein sets. This analysis yields an estimation of DMI enrichment within the dataset [34]. Enrichment for each viral group was first evaluated using the ELMi-Protein strategy, which works based on known DMIs in ELM database. The data were normalized by calculating the enrichment score, defined as the ratio of the predicted DMIs to the mean number of randomly expected DMIs:

$$Escore=\frac{{DMI}_{pred}}{{\mu DMI}_{rand}}$$

### 4.3. DMI Prediction in Different Viral Groups

ELM instances and domain information were then incorporated to increase the size of the network and discover new DMIs. First, known viral motif instances from the ELM database were used to predict DMIs in different viral groups using SLiMEnrich v1.5.1 through the ELMc-Protein (known viral SLiMs mapped to known human partner proteins via ELMs) and ELMc-Domain (known viral SLiMs mapped to Pfam-domain-containing human partner proteins) stringencies [34]. A false discovery rate (FDR) for individual DMIs was also estimated as the proportion of the predicted DMIs explained on average by random associations, using the mean random DMI count. A ratio of real DMIs was also calculated by subtracting random DMIs from the observed/predicted number of DMIs:

*DMI_real_* = *DMI_Obs_* − *DMI_rand_*

### 4.4. Host-Hijacked Proteins and Pathway Analysis

We aimed to investigate how different types of viruses hijack host proteins by analyzing their associations with different viral genomes: dsRNA, dsDNA, and ssRNA. To ensure a comprehensive analysis, we combined host proteins predicted to be hijacked in both ELMc-Protein and ELMc-Domain interaction datasets across all three viral types. Our goal was to understand whether certain host proteins are specifically targeted by one viral group or commonly targeted across multiple viral genome types. To do this, we categorized the combined list of hijacked host proteins into the following groups:

- Unique to a single viral group: proteins targeted only by dsRNA, dsDNA, or ssRNA viruses.
- Shared between two viral groups: proteins targeted by combinations of two viral types (dsRNA + dsDNA, dsRNA + ssRNA, or dsDNA + ssRNA).
- Shared across all three viral groups: proteins targeted by dsRNA, dsDNA, and ssRNA viruses.

By classifying host proteins in this way, we were able to evaluate the specificity and overlap in viral targeting strategies. This helps reveal whether certain host proteins are broadly exploited by diverse viruses or selectively hijacked by a particular class of viruses, offering insights into potential vulnerabilities in host–pathogen interactions. Moreover, a gene ontology pathway analysis of targeted host proteins was performed using the gProfiler [35] webserver, and pathways with FDR  <  0.05 were selected.

### 4.5. Cross-Validation of Predictions Using ELM Database

We then performed a cross-validation of the predicted DMIs by comparing them to known interactions from the ELM database, which contains validated viral–human SLiM-target domain pairs. The goal of this analysis was to assess the reliability of our predictions by determining the overlap with known SLiM-domain interactions reported in ELM.

To quantitatively evaluate the overlap between predicted and known interactions, we performed enrichment analysis using Fisher’s exact test. We used this test to compare our predicted interactions with those known in the ELM database, determining whether our predictions contained a higher proportion of interactions already validated in ELM compared to what would be expected by chance. In essence, we assessed whether the overlap between our predicted interactions and known SLiM-domain pairs in ELM was greater than what would occur randomly, helping to evaluate the biological relevance of our predictions.

### 4.6. Limitations

This study was based solely on the PHISTO database, and incorporating additional databases in future research would improve the reliability and scope of the findings. Another limitation was the high FDR of predictions, which increases the likelihood of false positives. As a result, individual results were not explored in depth. However, despite this limitation, we addressed an important question: how viral genomic composition influences motif mimicry and the mechanisms through which viruses hijack host cellular machinery. Reducing the FDR is crucial for enhancing the robustness and reliability of such analyses. This can be achieved by expanding available PPI data, particularly for underrepresented viral subtypes, or by categorizing viral proteins based on their roles in the viral life cycle. Additionally, implementing filtration steps for predicted DMIs could help lower the FDR, leading to fewer but more biologically meaningful predictions. By refining prediction methods and improving data quality, future studies can yield more precise and insightful results. Furthermore, future research could benefit from incorporating additional motif analysis tools such as iELM or ANCHOR and comparing outputs across these tools. A comparative analysis would strengthen confidence in predicted interactions and help address current limitations around validation.

## 5. Conclusions

This study investigated DMIs based on the genomic composition of various viral groups, with a focus on understanding how these viruses hijack host cellular machinery through motif mimicry. By evaluating the differences in PPI data across ssRNA, dsRNA, dsDNA, and ssDNA viruses, we were able to identify patterns in the interactions that each viral group predominantly forms with host proteins. The study predicted new DMIs, particularly for ssRNA and dsDNA viruses, which were found to be enriched with interactions, while dsRNA viruses showed limited interactions due to the small number of available PPI data. These findings highlight the role of viral genomic features in shaping viral-host interactions, enhancing our understanding of viral manipulation of host processes and providing insights into viral survival strategies that could inform future therapeutic research.

## Figures and Tables

**Table 1:** DMI Enrichment based on viral genomic composition.

Viral Group	vhPPIs ^1^	potDMI ^2^	predDMI ^3^	E-Score
dsRNA	76	2	2	19 **
ssRNA	18,932	17	6	6.7 **
dsDNA	12,755	27	19	11.6 **
ssDNA	321	0	0	NA

** *p*-value < 0.001. ^1^ Non-redundant vhPPIs. ^2^ Non-redundant number of all possible DMIs between a motif-containing protein and Pfam-domain-containing protein available in vhPPIs. ^3^ Non-redundant predicted DMIs.

**Table 2:** Number of unique and common host proteins targeted by different viral categories.

Group	Targeted Host Proteins
dsRNA	1
dsDNA	61
ssRNA	10
dsRNA + dsDNA	3
dsRNA + ssRNA	2
dsDNA + ssRNA	6
dsRNA + dsDNA + ssRNA	1

**Table 3:** Number of known interactions in ELM from our predicted datasets.

Viral Group	Known in ELM	Known (%)	Stringency
dsDNA	31	79.4% *	ELMc-Protein
dsRNA	2	66.6% *	ELMc-Protein
ssRNA	6	66.7% *	ELMc-Protein
dsDNA	21	16.5% *	ELMc-Domain
dsRNA	2	40.0% *	ELMc-Domain
ssRNA	6	31.5% *	ELMc-Domain

*p*-value < 0.005 = *.

## Data Availability

PPI data was retrieved from the PHISTO database, and DMI data is available as Supplementary Material.

## Supplementary Materials

The following supporting information can be downloaded at https://www.mdpi.com/article/10.3390/ijms26083674/s1.
